# Machine learning methods for histopathological image analysis: Updates in 2024

**DOI:** 10.1016/j.csbj.2024.12.033

**Published:** 2024-12-30

**Authors:** Daisuke Komura, Mieko Ochi, Shumpei Ishikawa

**Affiliations:** Department of Preventive Medicine, Graduate School of Medicine, The University of Tokyo, Tokyo, Japan

**Keywords:** Histopathology, Deep learning, Machine learning, Whole slide image, Computer-assisted diagnosis, Digital image analysis, Foundation model

## Abstract

The combination of artificial intelligence and digital pathology has emerged as a transformative force in healthcare and biomedical research. As an update to our 2018 review, this review presents comprehensive analysis of machine learning applications in histopathological image analysis, with focus on the developments since 2018. We highlight significant advances that have expanded the technical capabilities and practical applications of computational pathology. The review examines progress in addressing key challenges in the field as follows: processing of gigapixel whole slide images, insufficient labeled data, multidimensional analysis, domain shifts across institutions, and interpretability of machine learning models. We evaluate emerging trends, such as foundation models and multimodal integration, that are reshaping the field. Overall, our review highlights the potential of machine learning in enhancing both routine pathological analysis and scientific discovery in pathology. By providing this comprehensive overview, this review aims to guide researchers and clinicians in understanding the current state of the pathology image analysis field and its future trajectory.

## 1 Introduction

Histopathological examination is crucial in modern healthcare, particularly for cancer diagnosis. Traditionally dependent on pathologists’ expertise with microscopes, the field of pathology image analysis is now gradually incorporating digital pathology and advanced computational techniques to enhance the accuracy, efficiency, and reproducibility of pathological diagnosis [1], [2], [3], [4], [5]. Moreover, the computational analysis of vast numbers of histological images enables the discovery of biologically or clinically relevant knowledge [6], [7], [8].

**Fig. 1:**
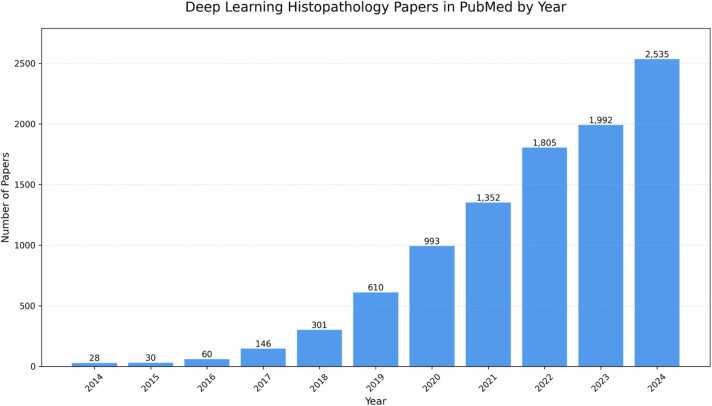
Number of research articles in PubMed with the keywords “deep learning” and “histopathology” between 2014 and 2024.

In 2018, we published a mini-review that provided an overview of digital pathology image analysis using machine learning techniques and important challenges to be addressed [9]. Since then, the field has witnessed remarkable progress, with numerous studies advancing both the accuracy and capabilities of pathological image analysis. The number of published articles on deep learning in histopathology has increased from 301 in 2018–2535 in 2024, i.e., a more than eightfold increase in just 6 years (Fig. 1). This surge in research activity has not only significantly improved the existing applications but also paved the way for novel applications that were not addressed in our previous review.

Despite these advances, several challenges persist, some of which were noted in our previous review. In this review, we present a comprehensive summary of computational pathology, with emphasis on the most impactful and novel approaches in these fields. We focus on challenges that are unique to or especially significant in pathological image analysis in order to provide valuable insights for pathologists and machine learning researchers new to this field.

In the following sections, we first discuss the key clinically and biologically important applications in computational pathology. We then explore specific challenges in this field and the solutions being developed to address these challenges.

## 2 Basic workflow of machine learning-based histopathological image analysis

Since 2018, deep learning approaches have become increasingly prevalent across all stages of pathological image analysis. Although deep learning methods were prevalent in 2018, there has been a continued shift toward more sophisticated deep neural network architectures for both feature extraction and downstream tasks.

**Fig. 2:**
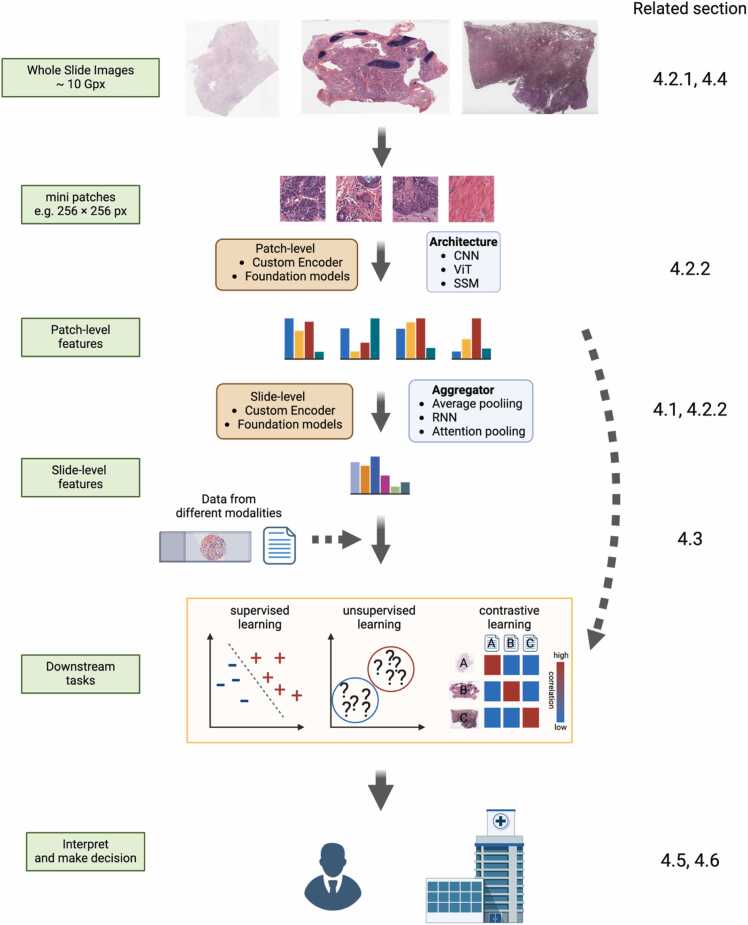
Workflow of histopathological image analysis with machine learning. Related sections in this review are shown in each process. Created in BioRender. Komura [33]https://BioRender.com/g69j665.

The basic workflow of pathological image analysis has remained largely consistent since 2018, primarily involving the extraction of fixed-size patches from whole tissue regions or regions of interest in whole slide images (WSIs) (4.1), followed by feature extraction from these patches (Fig. 2). Some studies have explored alternative approaches, such as the use of superpixels [10], [11] or constructing graphs [12], [13], [14], based on the detected tissue components. For tissue region detection, Otsu’s thresholding or adaptive thresholding method [15], [16] is commonly used to separate tissue from background. Regions of interest, such as tumor regions, are typically defined either by pathologists using specialized annotation tools [17], [18] or automatically through deep learning-based segmentation and classification methods [19], [20]. Various filtering techniques are applied to exclude patches containing blur or artifacts [21], [22], ensuring the quality of the extracted patches (4.4).

In the domain of feature extraction from these sampled patches, the use of vision transformers (ViTs) [23], [24] alongside conventional convolutional neural networks has increased due to their enhanced ability to capture contextual information. Methods to aggregate patch-level features into slide-level features have gained popularity [25], [26], [27], [28], [29] for slide-level tasks, such as prognosis prediction. Some approaches employ detection or segmentation techniques to identify and classify tissues, cells, or nuclei, followed by the creation of interpretable hand-crafted features, such as cell distribution, nuclear shape, and texture [30], [31]. These methods often involve constructing network structures with cells as nodes that are then used for classification and other analytical tasks.

The emergence of large-scale pretrained models, also known as foundation models pretrained on vast and diverse datasets of pathological images, has markedly advanced pathological image analysis [24], [27], [29], [32] (4.2.2). These models are used to extract patch- or slide-level features. After appropriate feature extraction, various analytical tasks common to image analysis are performed, such as supervised learning, unsupervised learning, multiple instance learning (also known as weakly supervised learning), and contrastive learning. In some applications, features from various modalities, such as text and transcriptome data, are integrated to learn the correlations between modalities or predict clinical information using multiple modalities (4.3). These tasks can be performed either as a separate step after feature extraction or integrated into an end-to-end training process with the feature extractor. Finally, the output might be visualized in an interpretable way (4.5), and a clinical decision may be made based on the result in clinical settings (4.6). Fig. 2.

## 3 Machine learning application in digital pathology

Machine learning techniques have enabled diverse applications in pathological image analysis, ranging from diagnostic support to novel biological discoveries. This section highlights the key applications that have demonstrated practical impact.

### 3.1 Computer-assisted diagnosis (CAD)

CAD systems use machine learning algorithms to assist pathologists in interpreting histopathological images. These systems primarily employ supervised learning approaches, which can be categorized into three main tasks as follows: classification, detection, and segmentation. In pathology, classification tasks involve several key applications, such as tissue categorization, including cancer subtypes [2], [3], [4], [5], [34], [35], [36], tumor grade assessment [2], [37], assessment of pathologic response to chemotherapy [38], and identification of primary cancer sites in cases of unknown origin [39]. Detection or segmentation tasks involve localizing specific pathological structures, such as metastatic foci in the lymph nodes [40], [41].

Although the basic concept of CAD has remained unchanged since 2018, recent studies have expanded significantly in scale, analyzing thousands of patients across multiple institutions [42], [43], [44], [45], [46], [47], [48]. These studies have not only facilitated more accurate predictions but also provided crucial validation of the robustness of CAD systems in settings that more closely resemble real-world clinical settings. Building upon these advances, a significant milestone in CAD is the FDA approval of clinically approved systems [49], [50], signaling their readiness for real-world healthcare integration.

Content-based image retrieval (CBIR) is a CAD technique that enables searching for images based on their visual content. At its core, CBIR operates by finding similar images through a nearest neighbor approach, comparing image features to identify matches with existing cases [51]. CBIR systems are especially valuable for rare cases [27]. When pathologists encounter rare tumors, they typically consult other pathologists or reference materials, such as books published by the World Health Organization. Instead, CBIR systems offer a powerful alternative, providing quick access to visually similar cases and associated diagnoses, assisting decision-making by pathologists. In patch-based CBIR, which was predominant until 2018, pathologists select diagnostically important regions within an image to use as queries [51], [52], [53]. This method allows for focused searches based on specific histological features or patterns that the pathologist deems crucial for diagnosis, leveraging their expertise in the search process. Patch-based systems are lightweight and versatile, making them applicable even to smartphone-captured images [51]. By contrast, WSI-based CBIR, a method that uses WSIs as queries, has recently emerged [27], [54], [55], [56], [57]. This process typically involves extracting slide-level features that capture global tissue characteristics and overall diagnostic information, enabling efficient retrieval of similar cases at the slide level. Both approaches have their merits. Although patch-based CBIR offers precision and efficiency, WSI-based CBIR provides a more holistic view of the tissue sample. The choice between these methods often depends on the specific diagnostic needs, available resources, and the nature of the pathological specimens under examination.

Pathologists observe pathological specimens to make diagnoses and then write reports. These reports contain information that could be considered the pathologist’s diagnosis itself, including histological findings related to tumor diagnosis, diagnostic basis, and results of immunohistochemical staining. Recently, attempts have begun to make artificial intelligence (AI) perform this task. This has been made possible largely due to the development of high-performance vision–language encoders and language decoders [27], [28], [58], [59], [60].

### 3.2 Predicting or discovering clinicopathological correlations

Pathological image analysis has evolved from basic diagnostic support to enabling precision medicine, particularly in oncology [61]. Since 2018, the field has seen rapid growth in both the sophistication of machine learning approaches and the scope of their applications, utilizing supervised learning methods to uncover novel correlations between histological patterns and clinical variables, such as prognosis, treatment efficacy, and genetic mutations from histological images [51], [62], [63], [64], [65], [66].

These analyses have direct practical applications in clinical settings. For example, predicting mutations from routine histological images can identify patients negative for mutations without expensive genetic testing, reducing healthcare costs [67], [68]. In prognostic prediction, incorporating model outputs as covariates may reduce the required number of enrolled patients in new anticancer drug development [69]. Treatment response prediction can help ensure that patients receive appropriate treatments, reducing the risk of suffering from side effects of ineffective treatments while enabling quicker transitions to more effective drugs [70], [71], [72]. For expensive treatments, such as immune checkpoint inhibitors, this approach could contribute to healthcare cost reduction by minimizing the wasteful use of costly medications [73], [74].

Beyond clinical applications, machine learning analyses of pathological images provide insights into cancer mechanisms and biology [6], [7], [8]. Developing interpretable models to translate computational findings into meaningful pathological understanding remains a key challenge, which we discuss in detail in 4.4. For example, Wulczyn *et al*. [6] developed a deep learning system to predict survival in colorectal cancer from histological images and introduced a clustering-based method to generate interpretable histological features that explain their model’s predictions. The authors identified a feature characterized by poorly differentiated tumor cell clusters adjacent to adipose tissue, which was associated with poor prognosis. Consequently, this study highlights the need for interpretable AI models to gain clinically relevant pathological insights.

### 3.3 Virtual staining

Traditional tissue staining methods, although essential for pathological diagnosis, can be time-consuming, costly, and require precious tissue samples. Virtual staining, which emerged around 2020, addresses these limitations by generating immunohistochemical or special stains from hematoxylin and eosin (H&E)-stained images [75], [76], [77] or predicting H&E or special stains from unstained specimens [78], [79], [80], [81]. This technology offers several practical benefits. It can reduce staining costs, preserve valuable specimens, and mitigate domain shift caused by staining variability. Additionally, it enables multiple staining patterns to be generated from a single tissue section, which is particularly valuable for small structures, such as glomeruli, where consecutive sections may not capture the same features. Another promising application is intraoperative rapid diagnosis, where virtual staining can enhance the quality of frozen section images by converting them to appear more like standard formalin-fixed paraffin-embedded sections [82]. Another interesting usage of virtual staining is that incorporation of virtually stained sections could improve the performance of downstream tasks, such as survival prediction [83].

From a machine learning perspective, virtual staining is implemented as a pixel-wise regression or image-to-image translation task. The field initially relied on supervised learning techniques but has increasingly adopted generative models, such as generative adversarial networks (GANs) [75], [84] and diffusion models [85], [86], which have shown remarkable progress since the early 2020 s. For example, de Haan *et al.* demonstrated the use of GANs for transforming H&E-stained kidney biopsy images into virtual special stains (Masson’s trichrome, periodic acid-Schiff, and Jones silver stain) [75], improving the preliminary diagnoses of non-neoplastic kidney diseases.

Despite these advances, it is crucial to understand the fundamental difference between actual staining methods and deep learning-based virtual staining. Although traditional staining relies on chemical reactions, deep learning-based virtual staining derives patterns from tissue morphology and texture. These distinct principles can lead to significant discrepancies between virtual and actual staining results [81], [87], [88], requiring careful validation in clinical applications.

## 4 Problems in histopathological image analysis

Histopathological image analysis presents unique technical challenges that distinguish it from other medical imaging domains. Since our 2018 review, some challenges have been partially addressed, whereas others have emerged or persisted. This section examines five critical areas as follows: very large image size, insufficient labeled images, multidimensional analysis, domain shifts, and interpretability. For each area, we discuss recent advances, current limitations, and promising research directions for the machine learning community.

### 4.1 Very large image size

The analysis of WSIs presents a fundamental computational challenge. Although a pathology specimen on a glass slide typically measures up to a few cm^2^, high-resolution slide scanners produce WSIs that can reach several gigapixels (Fig. 1a). To manage the massive images that exceed conventional memory capacity, researchers typically divide WSIs into smaller patches for analysis.

Since 2018, significant advances have been made in addressing the critical challenge of effectively combining information from individual patches to make slide-level decisions. Prior to these advances, early approaches used simple aggregation methods, such as majority voting and pooling operations [64], [89], [90], [91]. However, these methods had significant limitations—max pooling considers only the most prominent signals, potentially missing important contextual information, whereas average pooling dilutes important features by distributing equal weight to all regions, including nonrelevant areas, such as normal tissue, in cancer classification tasks.

The limitations of these simple approaches drove the development of more sophisticated aggregation methods, such as recurrent neural networks (RNNs) [2] and attention pooling [92], [93], [94], [95], [96], [97]. RNNs address the challenge of maintaining context while processing patches sequentially. The network builds understanding incrementally, updating its hidden state with each new patch while retaining relevant information from previous patches. However, RNNs face significant practical limitations—training difficulties due to vanishing or exploding gradients, limited long-term memory, and slow processing due to their sequential nature.

Attention pooling overcame several of these limitations by enabling parallel processing and direct modeling of the correlations between patches. This approach dynamically learns to identify and focus on the most diagnostically relevant regions while suppressing the less informative areas. However, its computational demands grow quadratically with the number of patches, creating challenges for large WSIs.

To address this, various strategies have been proposed that include creating multiresolution hierarchical structures [27], [98], [99], [100]. Hierarchical representation is a natural approach because pathologists typically begin with a low-magnification WSI to identify global structures and then transition to higher magnification images to identify fine-grained structures. For example, hierarchical image pyramid transformer [100] starts with 16 × 16 pixel images at 20 × magnification to capture cellular features. These cellular features were aggregated into 256 × 256-pixel regions representing cellular organization, with further aggregation into 4096 × 4096 tissue phenotypes and finally into WSIs. Feature aggregation is accomplished with the same mechanism using a per-instance multilayer perceptron, followed by a ViT and global pooling. LongViT [29] employs efficient dilated attention to capture correlations between distant patches. The advantage of this approach is a simple architecture that can be trained with efficiency in computation and memory while capturing both short- and long-range dependencies. The computation complexity of LongViT is O(Nd), which is much smaller than vanilla attention with $O(N^{2}d)$, where N is the number of patches and d is the hidden dimension.

Recently, state-space models, such as Mamba-2 [101] and Vision Mamba [102], [103], [104], have gained significant attention in the field due to their ability to capture longer-range interactions. These models can compress and retain important information similar to RNNs, but they operate selectively in a data-dependent manner. This selective nature means they can efficiently identify and retain only the most relevant information from the input data, which results in longer memorization. Additionally, the computation can be parallelized, which makes state-space models more computationally efficient than RNNs. Although the original Mamba architecture results in spatial discrepancies due to the limitations of 1D sequence processing, a Mamba derivative that processes a WSI in a 2D manner has been developed recently [105].

It is pertinent to mention that considering a wide range in some cases could be important not only for classification but also for detection and segmentation tasks [106], [107], [108], [109], [110], although this direction remains underexplored. For example, when pathologists encounter unfamiliar color tones or appearances, they may need to examine overall distribution to recognize cell morphology prototypes. Additionally, some structures cannot be classified based solely on their immediate surroundings, such as germinal centers in the lymph nodes, epithelioid macrophages, or well-differentiated hepatocellular carcinoma. This is because these structures may only become discernible when contrasted with other clear structures or surrounding tissues over a broader area. The importance of wide-range examination is also evident in tumor identification, where similarities between clearly defined tumor regions and other areas may inform diagnosis. This is particularly relevant because tumors are clonal, which means that tumor cells tend to have similar appearances to one another. Consequently, by examining a wider area, pathologists can identify patterns and similarities that may not be apparent when focusing on a small region, thus improving the accuracy of tumor diagnosis and classification.

### 4.2 Insufficiently labeled images

The scarcity of labeled histopathological images presents a fundamental challenge in the development of reliable machine learning models. This limitation stems from two key factors: 1) the time-intensive nature of expert annotation and 2) the privacy concerns surrounding medical data. To address this challenge, researchers have pursued two complementary approaches: 1) building comprehensive public datasets and 2) developing methods that can learn effectively from limited labeled data. In this section, we examine recent progress in both directions.

#### 4.2.1 Dataset

The development of robust pathological AI systems requires large-scale, diverse, and well-annotated datasets that capture the complexity of clinical diagnosis. Since 2018, the field has seen substantial growth in both the size and variety of public datasets (Table 1). The Cancer Genome Atlas continues to serve as the cornerstone resource for pathological image analysis, offering an extensive collection of WSIs across 32 cancer types. Its value stems from its comprehensive annotations, including diagnostic grades, prognostic information, and molecular data, such as somatic and germline mutations. This rich combination of imaging and molecular data enables researchers to investigate the correlations between morphological features and clinical outcomes. The versatility of The Cancer Genome Atlas has spawned numerous derivative datasets [51], [111], [112], where researchers have enhanced original data with additional annotations and preprocessing steps to address specific research questions in cancer pathology. Clinical Proteomic Tumor Analysis Consortium (CPTAC) provides another comprehensive multimodal dataset [113]. Recent years have seen the emergence of specialized large-scale datasets focused on specific cancer types. For example, IMP-CRS [114] offers > 5000 annotated slides for colorectal cancer classification, whereas PANDA [37] provides > 12,000 prostate cancer images with detailed Gleason grade annotations. These specialized datasets enable the development of highly accurate AI models for specific diagnostic tasks.

**Table 1:** Publicly available large-scale histopathology datasets.

Dataset name	# Samples	Annotation or additional modality	Disease or tissue
*Classification*			
TCGA [115], [116]	> 18,000 slides	Various clinical information on genome/transcriptome/epigenome pathology reports	32 cancer types
TCIA [117]	> 1400 slides (except for CPTAC)	Various clinical information	
CPTAC [113]	2059 slides	Various clinical information on genome/proteome/transcriptome	14 cancer types
PANDA [37]	12,625 slides	Gleason grade	Prostate cancer
IMP-CRS [114]	5333 slides	Grade (non-neoplastic, low/high grade lesions)	Colon tumor
DHMC [118]	563 slides	Renal cell carcinoma (RCC) subtypes	RCC
GTEx [119], [120]	25,380 slides	Tissue Bulk transcriptome	Normal
*Detection/segmentation*			
Camelyon [121]	1399 slides	Segmentation mask for the cancer region	Lymph node metastasis in breast cancer
BCSS	151. slides	> 20,000 segmentation annotations of tissue region by human (five tissue types)	Breast cancer
NuCLS [111]	125 slides	> 220,000 nuclei labels by humans (12 cell types)	Breast cancer
Lizard [122]	291 images	495,179 nuclei labels by humans (six cell types)	Colon cancer
MoNuSAC [123]	71 patients	> 46,909 nuclei labels by humans (four cell types)	Lung, prostate, kidney, and breast cancer
MIDOG+ + [124]	503 cases	∼12,000 cell labels for mitosis by humans	Seven cancer types
SegPath [125]	1583 patients (tissue microarray)	-Tissue segmentation labels for > 80,000 patches based on immunofluorescence (IF) images (three tissue types) - > 1500,000 nuclei labels and based on IF images (five cell types)	Tumors from 18 organs
*others*			
PathVQA [126]	5004 images	32,795 questions and answers for the image	Various diseases
Quilt-1M [127]	1000,000 images	Paired text	Various diseases
PLISM [128]	368,849 images from 46 WSIs	13H&E staining conditions with 13 imaging media (7 WSI scanners and 6 smarpthones)	48 tissue types
HEST−1k [129]	1108 WSIs	Paired spatial transcriptome and 60 million detected nuclei	25 organs

*All datasets contain formalin-fixed paraffin-embedded hematoxylin and eosin-stained images

The evolving needs of computational pathology have driven the creation of diverse specialized datasets that serve different purposes. PathVQA [126] is a visual question–answering dataset that is semiautomatically generated from textbooks and online digital libraries, comprising > 30,000 pathology images paired with relevant questions and answers. Quilt-1M [127] contains 1 million image–text pairs curated from various sources, including YouTube videos. These datasets can be used to create vision–language models for pathology (see 4.3.2 for details).

Another crucial development addresses the challenge of method generalization across different imaging conditions. The PLISM dataset [128] specifically tackles this issue by providing images of identical specimens captured under various staining methods and imaging devices—from professional slide scanners to smartphones. This resource enables researchers to develop and validate robust analysis methods that can perform consistently across various technical conditions—a critical requirement for real-world clinical applications.

The growing sophistication of AI applications in pathology has created a demand for more granular annotations. New datasets now provide detailed cell- and region-level annotations [111], [130], enabling the development of precise detection and segmentation models. These datasets mark a significant advance from earlier resources that provided only slide-level labels, allowing AI systems to identify specific structures and cellular features that pathologists use for diagnosis.

In addition to normal tissue features, researchers have recognized the importance of handling technical artifacts in pathological images. New specialized datasets document common imaging artifacts and preparation issues [131], providing essential training data for developing robust AI systems that can maintain performance despite image quality variations.

Dataset creation has evolved from purely manual annotation to a more efficient, multifaceted approach. One major advancement is the use of AI-assisted annotation, where deep learning models provide initial labels for expert refinement [111], [132], [133], [134]. This semiautomated approach, combined with crowdsourcing techniques and the involvement of medical students, along with pathologist verification, has greatly reduced the time and effort required for manual annotation while creating educational opportunities. Advances in annotation techniques that rely less on manual input have emerged. Immunohistochemistry or immunofluorescence staining highlights specific cell types or proteins, enabling the creation of more accurate and objective annotations with minimal manual labor [125], [135]. SegPath demonstrates the potential of automated annotation approaches for the semantic segmentation of H&E images. It uses immunofluorescence retaining as the ground truth to identify tissue and cell types, including epithelial tissues, various immune cells, smooth muscle tissues, and red blood cells, across 18 organ types [125]. The development of SegPath revealed specific limitations in manual annotation practices. Pathologists were found to report systematic biases in their annotations, such as overlooking plasma cells that lack typical cartwheel-shaped nuclei. The use of immunofluorescence data as a reference enables more consistent annotations that capture cellular diversity beyond the standard morphological criteria.

Multimodal datasets that combine different types of biological data have emerged as valuable resources. HEST-1k [129], which pairs pathological images with spatial transcriptomics data, has opened new avenues for integrating morphological- and molecular-level information in the analysis (see 4.3.3 for details). Such an approach not only offers high throughput but is also effective for annotating cells that even pathologists find challenging to label definitively [125].

Despite these advances, significant challenges persist in the field. Many datasets remain inaccessible or require direct requests to authors, often due to concerns over patient privacy. This limited access can subsequently impede the rapid progression of research. To address the challenge of data privacy while enabling collaboration, researchers have developed distributed learning approaches, such as federated and swarm learning [46], [47], [48]. Federated learning allows multiple institutions to train AI models collaboratively while keeping data local; only model updates are shared with a central server. Swarm learning achieves the same goal using blockchain technology without a central server. These approaches could enable institutions to contribute to public datasets while preserving patient privacy, because sensitive data never leaves its original location. The ultimate goal would be to leverage these technologies to create comprehensive public datasets and models that benefit research while respecting privacy regulations and patient confidentiality. This would allow institutions to contribute valuable data to advance research without compromising their patients’ privacy or violating data protection laws.

Changes in the diagnostic criteria over time present a unique challenge for pathology datasets. Although most machine learning approaches assume stable ground truth labels, medical knowledge and diagnostic standards evolve continuously. A clear example is the diagnosis of glioma, which has transitioned from purely morphological assessment to genetic mutation-based classification [136]. This evolution in diagnostic criteria creates practical challenges for dataset curation. The same histological image may receive different diagnoses at different time points, not due to errors but because of advances in diagnostic standards. To address this challenge, datasets must include both historical diagnostic context and updated classifications, allowing AI systems to understand and adapt to evolving medical knowledge.

#### 4.2.2 Minimal supervision and foundation models

In 2018, the significance of using pretrained models for constructing high-accuracy prediction models with limited labeled training data was highlighted. At that time, the use of ImageNet pretrained models was commonplace. Although such models provided useful feature extractors, they were not optimized for the unique characteristics of pathology images. Self-supervised learning (SSL) emerged as a more effective approach, allowing models to learn directly from large collections of unlabeled pathology images without requiring extensive manual annotations.

SSL in computer vision is a learning paradigm where the model learns meaningful visual representations from unlabeled images by solving pretext tasks that can be automatically generated from the input data itself. SSL is effective for pathology images because of the scarcity of expert annotations for extremely large WSIs. Two main approaches have proven effective: masked image modeling and contrastive learning. In contrastive learning, the model learns to recognize that different views of the same image should have similar representations while maintaining distinct representations for different images. To create these different views, researchers have applied various data augmentation methods as follows: geometric transformations, such as rotation and flipping, and pathology-specific modifications, such as color jittering and stain normalization [137]. These color augmentations are important in pathology because they help the model learn representations that are robust to stain variations across laboratories and scanning protocols. Masked image modeling reconstructs randomly masked portions of images, typically masking patches and using an encoder with a deep decoder for reconstruction. This approach has proven effective in learning rich visual representations. Recent approaches such as iBOT [138] and DINOv2 [139] take advantage of both contrastive learning and masked image modeling. By combining these methods, the models can learn local tissue features and global structural patterns. This combined approach has proven effective for pathology images, where both detailed cellular features and overall tissue architecture are important for diagnosis.

The availability of large pathology image collections, sometimes exceeding one million WSIs [140], has enabled the development of foundation models. These models leverage SSL techniques to learn from vast amounts of unlabeled data [24], [140], [141], [142], [143], [144], [145], [146]. The emergence of high-capacity architectures, particularly ViTs, has made it possible to utilize these large-scale datasets for model training. For example, UNI [24] was trained on > 100 million patches from 100,000H&E-stained WSIs using ViT-Large architecture. The more recent model Virchow2G [145] expanded the scale significantly, training on 3.1 million WSIs, including both H&E and immunohistochemistry images, using an even larger ViT architecture. Both UNI and Virchow2G were trained with DINOv2. These models offer flexibility in their application. Researchers can use them as feature extractors with simple linear classifiers as zero-shot learners using k-NN classification or as pretrained models for task-specific fine-tuning. This versatility allows adaptation to various pathology tasks while reducing the need for large amounts of labeled data.

Although most foundation models focus on analyzing small tissue patches, the need to understand entire slides has led to more comprehensive approaches. Prov-Gigapath [29], PRISM [28], and TITAN [27] are pioneered slide-level foundation models. Each model uses a different approach to combining information across an entire slide. Prov-Gigapath utilizes LongNet with dilated attention [147] to aggregate patch-level features into slide-level features, PRISM adopts Perceiver network [148], which can be considered a variant of RNN, for aggregation, and TITAN builds hierarchical representation [27]. These models can be valuable because many recent tasks involve slide-level predictions, including tumor subtype classification and genomic mutation prediction.

The growing availability of labeled pathology data has enabled a new generation of foundation models based on supervised learning. Because segmentation of tissue and cells is important in pathology, foundation models targeting segmentation, such as BiomedParse [149], SegAnyPath [150], and PAGET [33], have been recently developed, which can be used for diverse tissues and modalities. Some foundation models take advantage of related diagnostic tasks through multitask learning [151], [152], [153], [154]. For example, CHIEF [154] is trained for various slide-level prediction tasks, including cancer detection, tumor origin identification, biomarker prediction, and prognosis prediction, for various cancer types. The advantages of these models is that they can be used as feature extractors as well as potentially used for specific tasks, such as cancer detection and nucleus segmentation without fine-tuning.

Different foundation models often excel at capturing different aspects of pathological images. Research has shown that combining these complementary models through ensemble approaches can improve performance across various diagnostic tasks [146]. Building on this insight, COBRA [155] takes ensemble learning further by integrating features from multiple foundation models to create comprehensive slide-level representations. This approach combines the strengths of different models while maintaining computational efficiency through the Mamba-2 architecture [101].

### 4.3 Multidimensional analysis

**Fig. 3:**
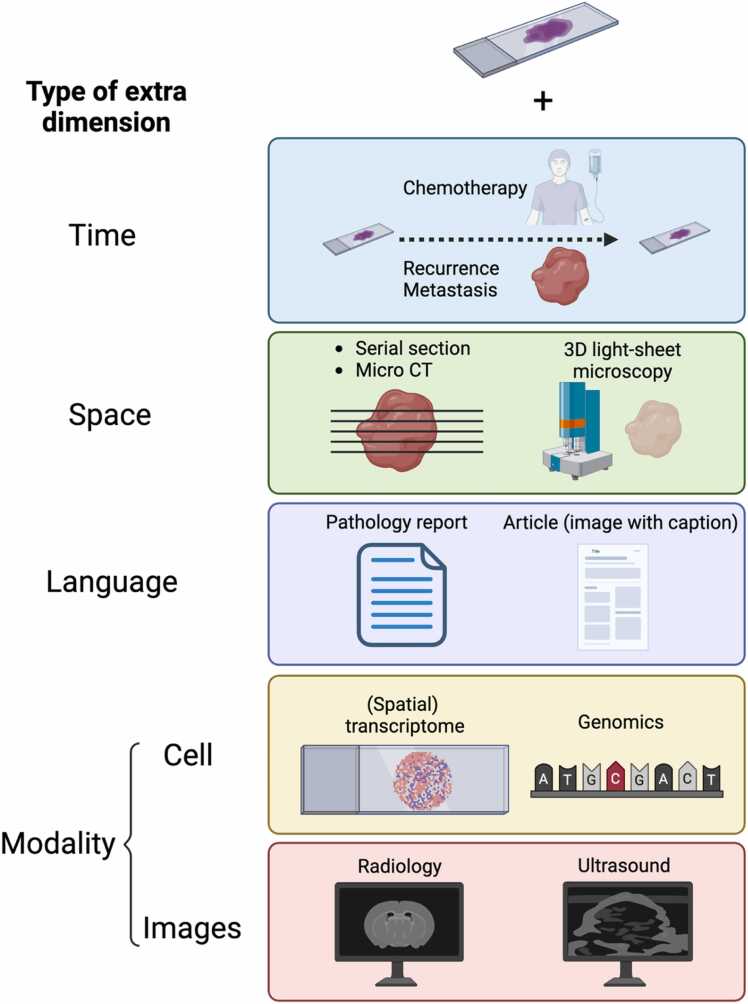
Various dimensions enhancing pathology image analysis. Temporal sequences, 3D histology, and multimodal integration for comprehensive diagnostic insights. Created in BioRender. Komura [33]https://BioRender.com/g69j665.

Pathological analysis increasingly incorporates multiple dimensions of data—temporal changes over disease progression, spatial correlations within tissues, and information from different analytical modalities (Fig. 3). Although analyzing temporal and spatial patterns within pathology images could build on established methods, combining data from different modalities, such as genomics, radiology, and pathology reports, requires new analytical approaches.

A combination of different types of biological data can enhance our understanding of disease processes. Each modality captures distinct aspects of pathology—images reveal tissue structure, genomics or transcriptomics identify molecular changes, and pathology reports provide diagnostic interpretations.

Multimodal data integration in pathology follows two main approaches. The first approach uses contrastive learning to align different modalities in a shared feature space. Each modality has its own encoder, but the encoders are trained to map similar content from different modalities to nearby points in the feature space. This alignment enables various applications, such as predicting molecular features from images [156], [157].

The second approach directly combines multiple modalities to predict clinical outcomes. This method requires careful consideration of how and when to merge different types of information [158], [159], [160], [161]. Early fusion strategies combine raw data immediately, whereas late fusion maintains separate processing paths until the final prediction stage. To manage the complexity of crossmodal interactions, models can employ specialized attention mechanisms that focus on specific modality pairs or process modalities hierarchically.

In the following sections, we will evaluate modality-specific applications and their associated challenges in pathological image analysis.

#### 4.3.1 Time and space

Temporal analysis in pathology aims to understand how diseases evolve, particularly in response to treatment. This understanding is crucial for several key clinical applications as follows: monitoring tumor response to therapy through changes in cellular morphology and immune cell infiltration; tracking the progression of chronic diseases, such as liver fibrosis; and evaluating transplant rejection to guide immunosuppression therapy. Another important application is the comparative analysis between primary tumors and their metastatic lesions, which can help verify metastatic origin and provide insights into tumor evolution and heterogeneity. However, this comparison presents unique challenges as the stromal components differ significantly between organs, necessitating methods to isolate and analyze tumor cell characteristics independent of the surrounding tissue microenvironment. Computational approaches like SegRep [162], which can extract features specifically from tumor cells while excluding stromal influences, may prove valuable for such analyses.

However, obtaining temporal data in pathology presents unique challenges. Although other medical imaging methods, such as CT, can easily track changes through repeated scans [163], [164], [165], pathological analysis requires tissue sampling, making frequent temporal measurements impractical and potentially harmful to patients. Once the tissue is removed for examination, that exact region can no longer be monitored over time. Instead, researchers must analyze different tissue samples from nearby regions, introducing variability that complicates temporal comparisons. These inherent constraints have resulted in limited research on the temporal analysis of pathological changes compared with other imaging modalities. As analytical methods and AI technologies advance, this field may see significant developments, particularly in techniques for comparing samples from different time points and locations.

Traditional pathology analysis relies on 2D tissue sections, which provide only a limited view of the complex tissue structures, and 3D analysis offers a more complete understanding of tissue architecture [166], [167], [168], [169], which is important for studying vessel networks, tumor invasion patterns, and cellular spatial correlations. Several technologies enable 3D tissue imaging, each with distinct capabilities. Serial sectioning reconstructs the 3D structure from multiple thin slices [170], [171], [172], microCT [173] provides detailed internal structure visualization, and 3D light-sheet microscopy with tissue clearing [174], [175] enables deep tissue imaging while preserving cellular detail. Song *et al*. performed pioneering work that analyzed 3D histological images using a deep learning framework specialized for 3D images [166]. They proved that patient prognostication using 3D tissue volumes outperforms 2D slice-based approaches in prostate cancer. Although these initial results are promising, the full potential of the 3D analysis methods will depend on accumulating large-scale 3D pathology datasets. As more institutions adopt 3D imaging technologies and contribute to data collection, we can expect further improvements in the accuracy and reliability of 3D pathology analysis.

#### 4.3.2 Language

Pathologists have developed a sophisticated language for describing tissue appearances over decades of practice. Recent AI models aim to leverage this expertise by learning from the text descriptions of pathological images. Several foundation models now use textual information from sources such as research articles and clinical reports to improve their understanding of pathological patterns. Notable examples are PLIP [157], CHIEF [154], CONCH [32], and PRISM [28], which fine-tune an SSL encoder using textual information, such as Twitter, PubMed research articles, and pathology diagnostic reports. PLIP and CHIEF adopt the CLIP framework [176], which uses dual encoders to project both images and text into a shared embedding space, and trains the model using image–text pairs to maximize similarity between correct image–text pairs while minimizing similarity for incorrect pairs. CONCH and PRISM adopt the Contrastive Captioner (CoCa) framework [177], which combines CLIP-style contrastive loss with a captioning loss using a vision–language decoder. A recent benchmarking study revealed that the CONCH image encoder demonstrates superior performance across multiple tasks compared with models trained exclusively on pathological images [178]. The success of these models may stem from their ability to focus on clinically relevant features while filtering out irrelevant image variations, similar to how pathologists learn to focus on diagnostic features. Traditional vision-based learning relies on data augmentation to teach models which features to ignore, such as color variations and rotations. Although this approach helps models become robust to technical variations, it provides no guidance about which features are clinically important. Leveraging textual information, which pathologists have developed a rich linguistic representation for describing pathological images over time, as supervisory information can potentially lead to improved feature extraction.

#### 4.3.3 Transcriptome

Transcriptomics data offer a new method to understand the correlation between tissue appearance and molecular function [179], [180], [181], [182]. Gene expression data provide detailed information regarding the cell types present in a region as well as their activation states and signaling pathways in a tissue. These molecular changes may be reflected, either directly or indirectly, in cellular and tissue morphology [51], [183]. This comprehensive molecular data may help capture subtle patterns and correlations in histology that may not be apparent through traditional human observation, enabling deep learning models to extract more nuanced and informative features from a biological system.

**Fig. 4:**
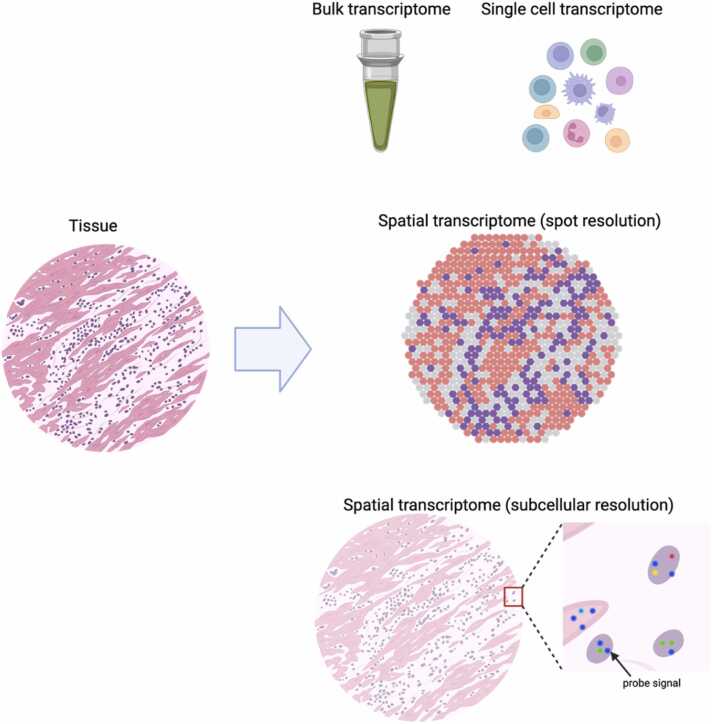
Various Transcriptome Technologies. Created in BioRender. Komura [33]https://BioRender.com/g69j665.

Transcriptome data can be obtained in three ways, each offering different insights into tissue biology (Fig. 4). Bulk transcriptomics measures average gene expression across an entire tissue sample, providing a comprehensive but spatially averaged view of cellular activity. Single-cell transcriptomics measures gene expression in individual cells, but spatial information is lost. By contrast, spatial transcriptomics preserves information about where genes are expressed within a tissue, enabling researchers to connect molecular activities with specific tissue regions. Different spatial transcriptome platforms offer varying spatial resolution capabilities: The original Visium [184] provides a moderate resolution of approximately 55 μm per spot, whereas the newer Visium HD [185] improves this to 2 μm per spot, although 10 × Genomics recommends using 8-μm spacing for optimal data quality and capture efficiency. Achieving even higher resolution, platforms such as CosMx [186], MERFISH [187], and Xenium [188] reach subcellular resolution (∼1 μm), enabling the precise localization of individual RNA molecules at single-cell resolution within the tissue context.

Analyzing the correlation between histology and transcriptome data presents different challenges depending on the type of transcriptome data available. For bulk transcriptomics, the primary challenge is the loss of spatial information, making it difficult to connect tissue-wide gene expression patterns with histological features. Although the tissue samples come from the same patient, they often represent different regions, requiring methods that can identify general patterns rather than exact correspondence.

Spatial transcriptomics offers a more precise mapping between gene expression and tissue structure, but similar issues persist. Researchers can either analyze consecutive tissue sections (one for H&E staining and another for transcriptomics) or perform both analyses on the same section. Consecutive sections may show different cellular patterns due to tissue heterogeneity, whereas using the same section can compromise staining quality due to the transcriptome analysis process. Recent platforms, such as Visium HD, have addressed these limitations by enabling transcriptome analysis after standard H&E staining, providing better alignment between molecular and visual data.

Transcriptome-based models serve two distinct purposes in pathology image analysis. The first aims to predict gene expression patterns directly from H&E images, potentially reducing the need for costly molecular testing. The second uses gene expression data as a form of supervision to help models learn more biologically meaningful features from images, similar to how molecular analysis guides pathologists’ understanding of tissue patterns.

These approaches have led to several modeling strategies. Some models use contrastive learning to align image and gene expression features in a shared mathematical space, enabling direct comparison between visual and molecular patterns. Others directly predict gene expression levels from images, either focusing on broad expression patterns or specific genes of interest. Each strategy has shown promise in different applications—contrastive methods excel at detecting general correlations between morphology and molecular states, whereas direct prediction methods are valuable for specific diagnostic tasks.

HE2RNA [189] and DeepPT [190] aim to predict bulk gene expression profiles from H&E-stained images, whereas the final aim of DeepPT is to predict cancer treatment response. Several methods predict the spatial transcriptome [180], [182], [191], [192], [193]. The development of spatial transcriptomics has led to more sophisticated approaches. HistoSPACE [180] trains an autoencoder for histology images, and its latent representation is attached to convolution and fully connected layers to infer spatial gene expression profiles. By contrast, TANGLE [179] performs crossmodal contrastive learning using bulk gene expression embeddings obtained through multilayer perceptron-based gene expression encoders. ConGcR and ConGaR [194] are contrastive learning-based models for integrating gene expression, spatial location, and tissue morphology for data representation and spatial tissue architecture identification utilizing graph convolution for gene expression and convolutional neural network for histological images. mclSTExp [195] and BLEEP [196] are intermediate approaches that perform crossmodal contrastive learning, and the models are also used for spatial transcriptome prediction.

#### 4.3.4 Other modalities

Although less commonly used compared with text and transcriptome data, integrating pathology images with other modalities can provide a comprehensive understanding of disease processes. This includes combining pathology images with (multiple) immunohistochemistry [197], [198], cytology [199], radiology images [200], bulk genomic data [201], [202], or epigenomic information [200]. IHC can identify specific proteins and markers, enhancing tissue analysis and disease classification. Cytology, particularly from biopsies, complements pathology by offering additional diagnostic insights. Radiology images, such as CT and MRI scans, are invaluable for noninvasive visualization of internal structures and can help correlate histological findings with anatomical changes, especially in cancer. Bulk genomic data contribute to the understanding of the genetic basis of diseases, and epigenomic data adds further depth by revealing gene regulation mechanisms. Recently, foundation models for various modalities have been developed, such as DNA [203], [204], protein [205], [206], CT [207], [208], and MRI [209]. These models could be integrated within multimodal models and improve disease diagnosis, predict progression, and personalize treatment, moving toward more tailored and effective healthcare strategies.

### 4.4 Domain shifts and artifacts

**Fig. 5:**
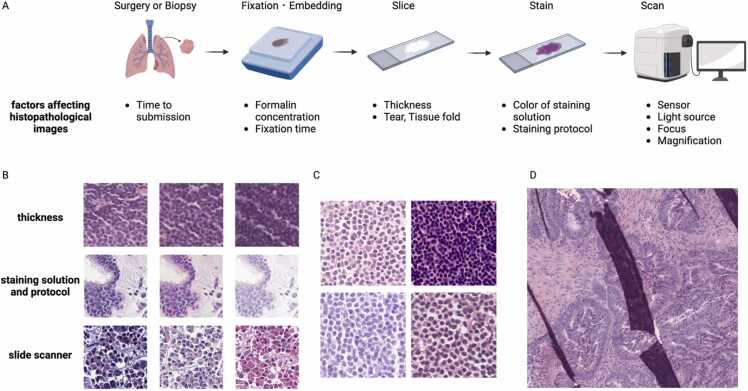
Variation in pathology images and artifacts. A) Various factors that affect image quality. B) Examples of images produced by factors such as tissue thickness, staining solution and protocol, and slide scanners. C) Tertiary lymphoid structures in gastric cancer slides from various institutions. D) Tissue fold artifact. Created in BioRender. Komura [33]https://BioRender.com/g69j665.

The journey from the tissue sample to the digital image involves multiple stages, each of which may contribute to the variations observed [210], [211]. Preanalytical factors, such as time to fixation, specimen size, sectioning, formalin concentration, and fixation duration, impact tissue quality, affecting image color and texture, and producing artifacts (Fig. 5). Section thickness during processing directly influences image appearance [212]. Even with standardized staining protocols, differences in stain manufacturers, lot, reagent age, and specific protocols may result in variations [128]. In addition, different scanner models and settings contribute to variations in the final digital image [128], [130]. These sources of variation present a significant challenge in pathology image analysis, especially when developing algorithms designed to generalize across institutions. The extracted features must reflect true biological differences rather than artifacts of image acquisition and processing.

The challenge of interinstitutional variability in pathology images aligns with the machine learning concepts of domain adaptation and generalization. Since 2018, several strategies have been proposed to address these issues. Data transformation, particularly stain normalization, remains the most common approach to standardize color distributions across images. The *de facto* standard methods transfer the estimated stain vector (H&E) of the source image to that of the target images using principle component analysis or non-negative matrix factorization [213], [214]. Although these core techniques remain unchanged, recent developments have focused on fast implementations, especially GPU-based libraries [215], [216], to improve processing efficiency.

Stain augmentation involves creating synthetic variations of existing images to expand the training dataset and improve model robustness. However, the degree of color variation often becomes an important issue. Therefore, setting an appropriate augmentation range is crucial. Excessive changes can undermine performance, whereas insufficient alterations may limit effectiveness [217]. As a result, advanced techniques have been proposed to address this issue, including meta-learning for the automatic optimization of augmentation parameters [218] and the selection of reference images from the distribution of other datasets [219], which aim to balance the creation of diverse, realistic variations with the preservation of the characteristics of the original image.

However, because color is not the only variable that requires normalization, data augmentation in the embedding space, rather than pixel space, may be one of the solutions [220]. Advanced techniques, such as cycle-consistent GANs (CycleGANs) [221] and diffusion models [222], have shown promise in translating images between domains. These techniques can be used to generate different images to address issues, such as fairness [223] and class imbalance [224]. Although these methods show promise, they have potential pitfalls. Image or feature translation techniques, for instance, run the risk of introducing hallucinations or artifactual features. This is particularly concerning in pathology, where subtle visual cues can have significant diagnostic implications. The challenge lies in distinguishing between variations due to different image acquisition protocols and those stemming from genuine biological differences, such as cell type variations or activation states. Incorrect modification of biologically relevant features could lead to decreased performance in certain applications.

Various methods have been developed to mitigate the generation of artifacts, such as detection and removal [21], [225], injection through data augmentation to enhance model robustness [226], and restoration using generative models [227]. In these approaches, the models and diverse training datasets containing a wide range of artifacts crucial are crucial. However, publicly available models and datasets are limited [21], [225], rendering their release desirable.

Although studies into adding these new dimensions are important, it is crucial to note that domain shift can be more pronounced in this type of analysis. In addition to the diversity in WSIs, a domain shift exists in clinical data, cytology, and radiological images, such as CT and MRI. The types of mutations and genes that can be recognized differ between whole genome and targeted sequencing. The diversity in spatial transcriptome measurement devices and protocols is likely greater than that in pathological images, resulting in significant variations in spatial resolution, detectable gene types, and patterns of noise and errors across platforms. A key challenge will be to determine how to robustly integrate this information. Additionally, as the number of modalities increases, the complexity of information grows, necessitating a large number of cases for the development of good machine learning models. However, because the cost and resources required to collect data for a single case increase simultaneously, the development of appropriate methods that could address these challenges will also be necessary.

### 4.5 Interpretability

Interpretability in pathological image analysis is important for several reasons. First, it enables pathologists or researchers to verify that the model captures the correct pathological phenomena, which is crucial for ensuring the reliability of automated diagnostic systems. Second, interpretable models can uncover new pathological indicators that might be useful for diagnosis. These discoveries could be incorporated into routine clinical practice by pathologists, expanding their diagnostic toolkit.

Although validating a model’s explanatory accuracy is relatively straightforward for diagnoses with known pathological features, discovering new diagnostic features without prior information presents significant challenges due to several factors. For example, pathological changes can occur at various scales, including individual cell types, cellular morphology, multicellular structures, spatial correlations between cells and structures, and alterations in noncellular components. Typically, it is not merely the individual features but their combinations that are diagnostically significant, which in turn increase the complexity of interpretation. Therefore, it is a significant challenge to present this multifaceted, complex information in a way that is understandable and actionable for human pathologists.

Since 2018, there has been an increasing trend in tasks where discriminative features are not well-known, such as when predicting genetic mutations. However, the development of explainable AI models has not advanced sufficiently to accommodate the trend.

**Fig. 6:**
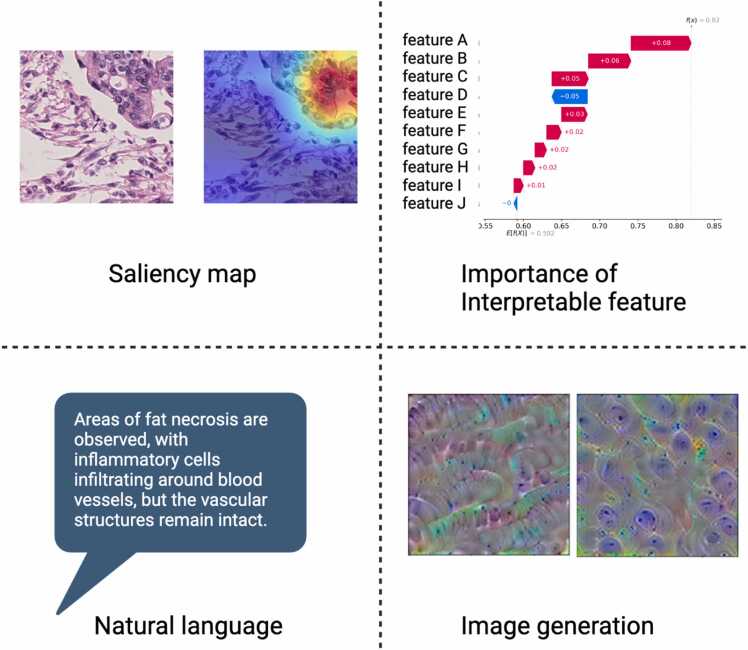
Approaches to interpretable artificial intelligence models.

The current approaches to interpretability in pathological image analysis can be broadly categorized into several types (Fig. 6). The first category includes post hoc methods, such as GradCAM [228], [229], integrated gradients [230], and attention, which highlight the regions that contributed significantly to the model’s decision. Although these methods can work with high-accuracy models, they often struggle to adequately represent complex pathological phenomena. To tackle this problem, MRAN [231] and E^2^-MIL [232], for instance, proposed a multilevel attention mechanism to identify both significant local and global structures in the WSI classification.

The second category involves inherently interpretable models, in which interpretable features are designed from the outset and used to build the classification model. The contributing features are then presented as the interpretation [233], [234]. Some of these approaches rely on accurate tissue or nucleus detection or segmentation models [235], [236] because they depend on information pertaining to tissue distribution, cell position, shape, and texture. Therefore, it is essential to introduce higher precision techniques that can accurately detect various components of tumor tissue. Another challenge in this approach lies in the appropriate design of features that can represent the full complexity of the pathological phenomena.

The third category involves image generation techniques. These include methods such as activation maximization [237], which generates tissue images that maximize the prediction probability for a given class, or approaches using generative models, such as CycleGAN or diffusion modes, to generate counterfactual images [238], [239], [240]. For example, Žigutytė *et al*. developed a method using a diffusion autoencoder to generate counterfactual pathology images across different types of cancer, including colorectal, liver, lung, and breast. Their method can generate images that show varying combinations of two classifications—for example, blending normal and cancerous tissue, squamous cell carcinoma with adenocarcinoma, or microsatellite instability-high with non-microsatellite instability-high cases [240]. Unlike these approaches, HIPPO [241] iteratively exchanges patches to generate a counterfactual patch set based on the classifiers output in order to capture important regions for classification.

Although these methods do not directly explain the decision-making process and rely heavily on the interpretive skills of pathologists, they can extract findings that go beyond specific image regions or verbalized features.

Another emerging and promising approach in this field is the use of vision–language models, such as PathAsst [242] and PathChat [60], which represent images through natural language descriptions. This method leverages the power of large language models to provide detailed, contextual descriptions of pathological images. By translating visual information into textual form, these models can bridge the gap between complex image data and human-interpretable insights. This approach depends on how pathologists traditionally communicate their findings and could therefore facilitate a more intuitive interaction with AI-assisted diagnostic systems. Notably, the ability of these models to identify novel features or patterns that have been unrecognized by pathologists remains to be validated. Moreover, these models can potentially generate hallucinations, producing plausible but inaccurate descriptions of pathological features. Although its potential is significant, careful evaluation is required to ensure both the reliability of these language-based interpretations and their ability to extend beyond known pathological knowledge, potentially contributing to new discoveries in the field.

### 4.6 Decision-making in pathological diagnosis

Most pathology AI technologies aim to make diagnoses or predictions from pathological images. However, the actual process of pathological diagnosis is significantly more complex. Additional tests, such as immunohistochemistry or FISH, may be necessary to narrow down the diagnosis. In some cases, it is important to review past specimens to track changes over time. Given this context, there is a growing need for AI technologies that can indicate additional tests or highlight important information to confirm diagnoses [60]. Such decision-making processes require not only histological knowledge but also consideration of a hospital’s medical resources and related costs as well as past clinical tests and medical records. In addition, it is crucial to engage in thorough discussions with other clinicians to gather diverse perspectives and expertise, ensuring a comprehensive approach to patient care and treatment planning. Therefore, a wide range of medical knowledge beyond diagnostic pathology is necessary [243]. Future research should focus on seamlessly integrating AI-assisted analysis tools into clinical pathology workflows to complement and enhance, rather than replace, human expertise. AI agents that can access various data sources and tools to automatically perform tasks based on text instructions are becoming increasingly popular, including in the field of pathology, as exemplified by systems such as Judith [244]. By addressing these challenges and exploring new directions, digital pathology image analysis can significantly impact clinical practice, enhance our understanding of disease mechanisms, and ultimately improve patient care.

In actual clinical settings, treatment decisions and other choices are made by integrating data from multiple modalities. In the future, it is desirable to develop decision support systems that integrate multimodal information to assist in decision making.

## 5 Summary

This review discusses various challenges and approaches in pathological image analysis. Since 2018, numerous methodologies have been developed, showing clear gradual improvement in the field. However, the emergence of new modalities, such as spatial transcriptomics, has expanded the applications of pathological image analysis, consequently bringing new technical challenges to the forefront.

Although research in 2018 primarily focused on basic research aspects, recent years have seen an increase in studies oriented toward clinical applications. This shift has raised the bar for the required accuracy levels. Although some challenges remain unresolved, the technology continues to evolve.

The review concludes that the contribution of pathological image analysis technologies to diagnosis, prediction, and treatment is expected to increase.

## Funding

This study was supported by JSPS KAKENHI Grant-in-Aid for Scientific Research (B) under Grant Number 21H03836 to D.K, The AMED Practical Research for Innovative Cancer Control under grant number JP 24ck0106873, and The AMED Practical Research for Innovative Cancer Control under grant number JP 24ck0106904 to S.I.

## CRediT authorship contribution statement

**Mieko Ochi:** Writing – review & editing, Visualization. **Shumpei Ishikawa:** Writing – review & editing, Supervision. **Daisuke Komura:** Writing – original draft, Visualization, Investigation, Conceptualization.

## Author contributions

Conceptualization: DK; Investigation: DK; Supervision: SI; Visualization: DK, MO; Writing-original draft: DK; Writing-review & editing: MO, SI.

## Declaration of Generative AI and AI-assisted technologies in the writing process

During the preparation of this work, the authors used Claude AI to improve language. After using this tool/service, the authors reviewed and edited the content as needed and take full responsibility for the content of the publication.

## Declaration of Competing Interest

The authors declare no competing interests.
